# Information Bottleneck for Estimating Treatment Effects with Systematically Missing Covariates

**DOI:** 10.3390/e22040389

**Published:** 2020-03-29

**Authors:** Sonali Parbhoo, Mario Wieser, Aleksander Wieczorek, Volker Roth

**Affiliations:** Department of Mathematics and Computer Science, University of Basel, Basel CH 4051, Switzerland; mario.wieser@unibas.ch (M.W.); aleksander.wieczorek@unibas.ch (A.W.); volker.roth@unibas.ch (V.R.)

**Keywords:** information bottleneck, mutual information, causal effect, average treatment effect, confounding, systematically missing, sufficient covariate, healthcare

## Abstract

Estimating the effects of an intervention from high-dimensional observational data is a challenging problem due to the existence of confounding. The task is often further complicated in healthcare applications where a set of observations may be entirely missing for certain patients at test time, thereby prohibiting accurate inference. In this paper, we address this issue using an approach based on the information bottleneck to reason about the effects of interventions. To this end, we first train an information bottleneck to perform a low-dimensional compression of covariates by explicitly considering the relevance of information for treatment effects. As a second step, we subsequently use the compressed covariates to perform a transfer of relevant information to cases where data are missing during testing. In doing so, we can reliably and accurately estimate treatment effects even in the absence of a full set of covariate information at test time. Our results on two causal inference benchmarks and a real application for treating sepsis show that our method achieves state-of-the-art performance, without compromising interpretability.

## 1. Introduction

Reasoning about the effects of an intervention is a key question across many applications such as healthcare [1,2], finance [3] and public policy [4,5]. In general, making such predictions on the basis of observational data containing only past actions, covariates and their outcomes is challenging, since we do not have access to the precise mechanism that led to a particular action choice. Specifically, the actions observed in the data may be determined by variables that also impact the outcome, resulting in confounding that otherwise biases predictions if unaccounted for (e.g., socioeconomic status may dictate what kinds of treatments a patient can afford and also affect their overall outcomes) [6]. Correcting for such confounding is thus crucial when estimating the effects of an intervention. The problem is especially more challenging where a set of measurements is missing or systematically missing for some patients at test time. Systematic missingness is a structured missingness where a fixed set of variables is unavailable for a proportion of the data of interest, often as a result of resource limitations at a particular time. It is a frequently occurring problem in many application areas: For instance, a doctor treating patients with HIV may have access to both the genotype and phenotype data for a particular study group of patients, but have a potentially larger group of patients outside the study group, for whom genotype data are unavailable as a result of the medical costs associated with genotyping. Here, estimating treatment effects for these patients requires integrating over all the missing variables—an infeasible task in high-dimensional settings.

A naive strategy to address the problem of systematic missingness in the data is simply to remove those features that are missing at test time, from the set of training data entirely and use a model trained only on the reduced space of features to infer treatment effects. However, this approach discards information that may be relevant in the training data for inferring outcomes. Alternatively, one might use multiple imputation or other imputation-based techniques to first impute the incomplete dimensions for the same purpose [7,8]. These methods work by generating a copy of the dataset in which the missing values are computed on the basis of an imputation model. The procedure is repeated several times in multiple imputation such that variations in the missing data can be accounted for. Unfortunately in high-dimensional settings with systematic missingness, imputation-based solutions can have large inaccuracies, particularly if many dimensions are missing. As a result, several other approaches have been developed to account for data missingness during training by additionally assuming for instance, hidden confounding. These methods typically try to build try to build a joint model on the basis of noisy representatives or proxies of confounders (see for instance [9,10,11,12]). However in high-dimensional-settings, it is unclear what these representatives might be or whether our data meet such assumptions. Importantly, regardless of these assumptions none of these approaches addresses systematic missingness at test time.

**Figure 1:**
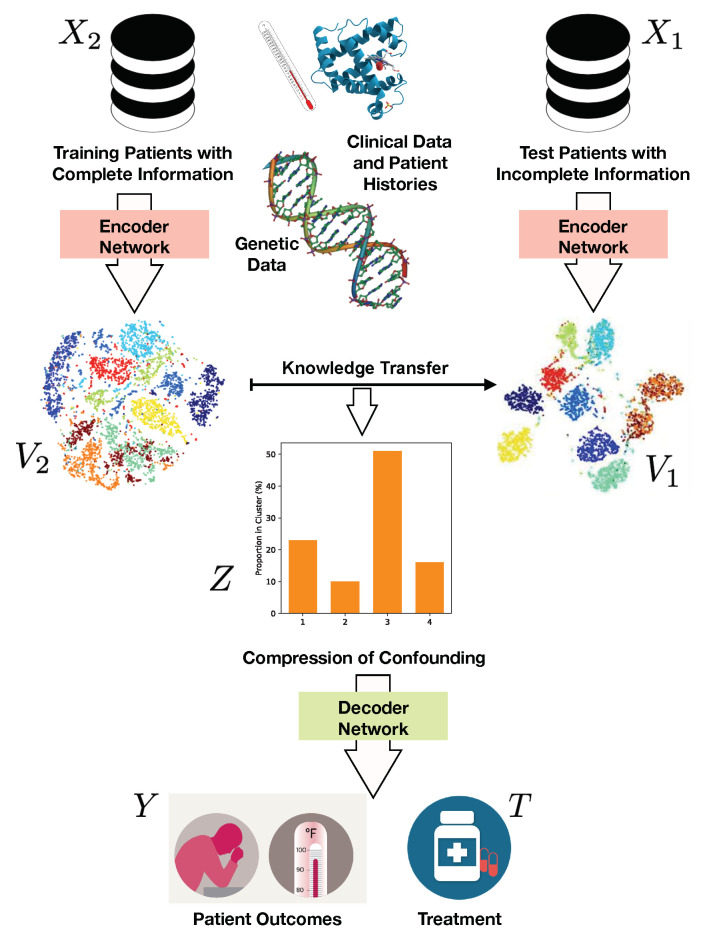
Graphical overview of our approach. We learn a discretised low-dimensional representation of confounding using the Information Bottleneck. As a result, we learn equivalence classes among the data such that we can use this information approximate the treatment effect for cases where data are systematically missing at test time.

A more natural approach would be to assume one could measure everything that is relevant for estimating treatment effects for a subset of the patients, and attempt to transfer this distribution of information to a potentially larger set of test patients. However, this is a challenging task given the high dimensionality of the data that we must condition on in order to infer treatment effects. In this paper, we address the problem from a decision-theoretic perspective of causal inference. Our goal is to reliably estimate treatment effects where data are systematically missing at test time. To do so, we work under the common assumption of strong ignorability. Our approach is based on the Information Bottleneck (IB) method [13,14] that was originally introduced as a compression technique to compress information about a random variable *X* while preserving relevant information about a different random variable *Y*. Here, we use the IB to learn a sufficient reduction of the confounding information for inferring treatment outcomes. A graphical overview of our approach is provided in Figure 1.

### Paper Contributions

Our contributions may be summarised as follows:

- First, we develop a method based on the Information Bottleneck to learn a compressed and interpretable representation of confounding.
- Based on this representation, we learn equivalence classes among patients such that the causal effect for a specific patient can be approximated using the specific causal effect of the subgroups.
- We subsequently transfer this information to a set of test cases where data is systematically missing at test time.
- We demonstrate on two causal inference benchmark data sets and a real world application for treating sepsis that our approach outperforms existing approaches.

The organisation of the remainder of the paper is as follows. Section 2 briefly reviews related work, while Section 3 introduces our approach to inferring treatment outcomes using the Information Bottleneck principle. In Section 4, we present our results on two popular causal inference benchmarks and two real world applications. Section 5 concludes the work with final remarks, and outlines directions for future research.

## 2. Related Work

A large amount of research in machine learning is targeted towards causal discovery with the aim of learning the underlying causal graph from observational data (e.g., [15,16]). We instead focus on causal deduction where the causal graph is known, and the task is rather to quantify the treatment effect of one variable on another. In this vein, many existing works have been dedicated to counterfactual reasoning and deep latent variable models. Since our key methodological contribution is based on a deep-variant of the information bottleneck, we discuss these and other related models in this section.

### 2.1. Potential Outcomes and Counterfactual Reasoning

Counterfactual reasoning (CR) has drawn large attention, particularly in the medical community. Counterfactual models are essentially rooted in causal inference and may be used to determine the causal effects of an intervention. These models are formalised in terms of potential outcomes [17,18,19]. Assume we have two choices of taking a treatment *t*, and not taking a treatment (control) *c*. Let $Y_{t}$ denote the outcomes under *t* and $Y_{c}$ denote outcomes under the control *c*. The counterfactual approach assumes that there is a pre-existing joint distribution $P(Y_{t},Y_{c})$ over outcomes. This joint distribution is hidden since *t* and *c* cannot be applied simultaneously. Applying an action *t* thus only reveals $Y_{t}$, but not $Y_{c}$. In this setting, computing the effect of an intervention involves computing the difference between when an intervention is made and when no treatment is applied [20,21]. We would subsequently choose to treat with *t* if,

(1) $$\begin{array}{c} E\left[L\left(Y_{t}\right)\right]\leq E\left[L\left(Y_{c}\right)\right] \end{array}$$

for loss *L* over $Y_{t}$ and $Y_{c}$ respectively. Potential outcomes are typically applied to cross-sectional data [22,23] and sequential time settings. Notable examples of models for counterfactual reasoning include Johansson et al. [6] and Bottou et al. [24]. Specifically, Johansson et al. [6] propose a neural network architecture called TARnet to estimate the effects of interventions. Similarly, Gaussian Process CR (GPCR) models are proposed in Schulam and Saria [22,23] and further extended to the multitask setting in Alaa and van der Schaar [2]. Off-policy evaluation methods in reinforcement learning (RL) offer another perspective for reasoning about counterfactuals, and have been extensively explored to estimate the outcomes of a particular policy or series of treatments based on retrospective observational data (see for example Dudík et al. [25], Thomas and Brunskill [26], Jiang and Li [27]). Unlike each of these techniques though, we focus specifically on estimating treatment effects where covariates are systematically missing at test time.

### 2.2. Decision-Theoretic View of Causal Inference

The decision theoretic approach to causal inference focuses on studying the effects of causes rather than the causes of effects [28]. Here, the key question is what is the effect of the causal action on the outcome? The outcome may be modelled as a random variable *Y* for which we can set up a decision problem. That is, at each point, the value of *Y* is dependent on whether *t* or *c* is selected. The decision-theoretic view of causal inference considers the distributions of outcomes given the treatment or control, $P_{t}$ and $P_{c}$ and explicitly computes an expected loss of *Y* with respect to each action choice. Finally, the choice to treat with *t* is made using Bayesian decision theory if,

(2) $$\begin{array}{c} E_{Y∼P_{t}}\left[L\left(Y\right)\right]\leq E_{Y∼P_{c}}\left[L\left(Y\right)\right]. \end{array}$$

Thus in this setting, causal inference involves comparing the expected losses over the hypothetical distributions $P_{t}$ and $P_{c}$ for outcome *Y*. In this paper, we formulate our model in terms of the decision-theoretic perspective of causal inference.

### 2.3. Estimating Treatment Effects with Missing Covariates

A sizeable amount of work has previously been done on causal inference with missing data. In particular, [29] propose using a latent mixture model to perform multiple imputation in order to estimate treatment effects and compute propensity scores. Similarly, Cham and West [30] present an empirical example of the performance of propensity score estimation methods when adapted to missing covariates. More recently, Kallus et al. [31] performed a low-rank matrix factorisation on a noisy set of covariate matrices to deduce a set of confounders based on which one can infer treatment effects. The approach is general enough to adapt to scenarios where covariates are missing at random and can be used as a preprocessing step for other bias correction techniques such as propensity reweighting. Unlike these approaches, we make use of the IB criterion to adjust for the effects of confounding and learn treatment effects. We specifically focus on a different type of missingness, namely systematic missingness at test time.

### 2.4. The Information Bottleneck

Let the Kullback Leibler divergence between two probability distributions *p* and *q* be denoted as $D_{KL}\left(p\left(X\right)||q\left(X\right)\right)=E_{p(X)}\log\frac{p(X)}{q(X)}$ which is non-negative. Let I(X;Z) denote the mutual information between *X* and *Z* where $I\left(X;Z\right)=D_{KL}(p\left(X,Z\right)||p\left(X\right)p\left(Z\right)$. Given two random variables *X* and *Y*, the IB method [13] searches for a third random variable *Z* that, while compressing *X*, retains information about *Y*. The resulting problem is defined as:

(3) $$\begin{array}{c} \min_{p(z|x)}I\left(X;Z\right)-\lambda I\left(Z;Y\right), \end{array}$$

where λ is a parameter that trades off the degree of compression of *X* with preservation of *Y*. The classical IB method assumes that variables satisfy the Markov relation Z−X−Y, i.e., that given *X*, *Z* and *Y* are conditionally independent. In its classical form, the IB principle is defined only for discrete random variables. However, in recent years, multiple IB relaxations and extensions, such as for Gaussian [32] and meta-Gaussian variables [33], have been proposed. Among these extensions, is the latent variable (deep) formulation of the IB method that first appeared in [34], and then in [14,35]. Ref. [34] demonstrate how optimal representations of deep neural networks can be expressed in terms of information dropout based on the IB method, while in parallel [14] consider variational lower bounds on the information bottleneck problem for optimisation. The latter requires an analytically solvable form of the lower bound in order to learn an optimal representation, while [34] propose methods to learn this representation without such forms. Importantly, both [34] and [14] assume the model adheres to the data generating process described by structural equations of the form,

(4) $$\begin{array}{c} z=f\left(x\right)+\eta_{z}, \end{array}$$

(5) $$\begin{array}{c} y=g\left(z\right)+\eta_{y}, \end{array}$$

where $\eta_{y},\eta_{z}$ are noise terms independent of *Z* and *X* respectively. These equations give rise to a conditional independence relation that is different to the classical formulation, i.e., X−Z−Y where given *Z*, *X* and *Y* are conditionally independent. While both independences from the classical IB and latent variable formulation cannot hold in the same graph, Wieczorek and Roth [36] show that it is possible to lift the original IB assumption in the context of the deep IB by optimising a lower bound on the mutual information between *Z* and *Y*. In this paper, we use the latent variable formulation of the IB and extend the formulation to deduce treatment effects where covariates are systematically missing at test time.

### 2.5. Latent Variable Models

Various probabilistic modelling techniques have been developed for similar tasks; for instance, Ref. [37] build implicit causal models for application to genome-wide association studies. Similarly, deep latent variable models have also received remarkable attention and been applied to a variety of problems. Among these, variational autoencoders (VAEs) employ the reparameterisation trick introduced in [38,39] to infer a variational approximation over the posterior distribution of the latent space p(z|x). Important work in this direction includes Kingma et al. [40] and Jang et al. [41]. Most closely related to the work we present here, is the application of VAEs in a healthcare setting by Louizos et al. [12]. Here, the authors introduce a Cause-Effect VAE (CEVAE) to estimate the causal effect of an intervention in the presence of noisy proxies. It has been shown that there are several close connections between the VAE framework and the latent variable formulation of the IB [14]. The latter is essentially a VAE where *X* is replaced by *Y* in the decoder. Finally, Kaltenpoth and Vreeken [42] propose using Probabilistic PCA to deduce a set of confounders and reason about treatment effects. Unlike both of the aforementioned methods however, our approach considers a different causal graph and specifically uses the IB method to learn a compressed representation of the covariate information for reasoning about treatment effects where covariates are systematically missing at test time.

## 3. Method

In recent years, there has been a growing interest in the connections between the IB principle and deep neural networks [14,35,43]. In this section, we present a model using the IB principle, for estimating the effects of an intervention in scenarios where covariates are systematically missing during testing. Throughout the rest of this paper, we will refer to our approach as a Cause–Effect Information Bottleneck (CEIB). Specifically, we combine the non-linear expressiveness of neural networks with the IB criterion to learn a suitable representation of confounding, which we use to make inferences about treatment effects. Our model thus consists of three main steps:

1. First we perform a low-dimensional compression of covariate information using the Information Bottleneck to identify a discretised representation of confounding.
2. We use the representation to learn equivalence classes among patients, such that the treatment effect for a specific patient can be approximated using the treatment effect of the equivalence class associated with them.
3. Finally, we transfer this information to a set of test cases where data are systematically missing at test time.

In what follows, we present our model and interpret our results from the decision-theoretic perspective of causal inference [28]. We elaborate on each of above steps in the next sections. The majority of the work we present here is based on Parbhoo [44].

### 3.1. Quantifying Causal Effects

Our goal in this paper is to make predictions about the causal effects of an intervention by estimating the the Average Causal Effect (ACE) (or average treatment effect) [28] of an intervention *T* on outcomes *Y*. In the interventional regime or setting, this is possible by actively intervening and assigning treatments, or by performing an experimental study, e.g., randomised control trial to directly measure the effects of introducing an intervention on subjects. Let $F_{T}=0$ and $F_{T}=1$ denote the interventional setting where we actively assign or intervene on *T*. Here, the ACE is given by simply measuring the difference in the outcomes of patients assigned to each intervention $F_{T}=0$ and $F_{T}=1$. That is, the ACE is formally given by

(6) $$\begin{array}{cc} ACE: & =E\left[Y|F_{T}=1\right]-E\left[Y|F_{T}=0\right]. \end{array}$$

In general, however, it is not always possible to perform interventions to reason about treatment effects directly, due to the costs and risks that may be incurred. As a result, we must frequently rely on observational data to make inferences about treatment effects. The hallmark of learning from observational data is that the actions observed in the data depend on variables which might also affect the outcome, hence resulting in confounding. In the observational setting, the ACE can be written as:

(7) $$\begin{array}{cc} ACE: & =E\left[Y|T=1,F_{T}=\emptyset \right]-E\left[Y|T=0,F_{T}=\emptyset \right], \end{array}$$

where $F_{T}=\emptyset$ denotes the fact that we no longer have control over the intervention/treatment assignment and simply observe *T* as a random variable. Importantly, because of the influence of confounding, the ACE in Equations (6) and (7) are in general, not equal unless we assume ignorable treatment assignments or assume the conditional independence relation $Y⊥\;⊥F_{T}|T$. This assumption expresses that the distribution of Y∣T is the same in the interventional and observational regimes, and is equivalent to the setting where there is no confounding.

For the more general case where confounding is present, the treatment assignment $F_{T}$ may only be ignored when estimating *Y* if provided a sufficient covariate, *Z*, and *T* [28]. Here, *Z* is a sufficient covariate for the effect of *T* on outcome *Y* if $Z⊥\;⊥F_{T}$ and $Y⊥\;⊥F_{T}|\left(Z,T\right)$. In this case, it can be shown by Pearl’s backdoor criterion [20] that the ACE may be defined in terms of the Specific Causal Effect (SCE),

(8) $$\begin{array}{c} \begin{array}{cc} ACE: & =E[SCE\left(Z\right)|F_{T}=\emptyset ] \end{array} \end{array}$$

where

(9) $$\begin{array}{c} \begin{array}{cc} SCE(Z): & =E\left[Y|Z,T=1,F_{T}=\emptyset \right]-E\left[Y|Z,T=0,F_{T}=\emptyset \right]. \end{array} \end{array}$$

Overall, the SCE may be viewed as an equivalent of the ACE, restricted to a subspace of the population with a certain value of Z=z. In this paper, we identify a low-dimensional representation of confounding using the Information Bottleneck Principle. We discretise this representation such that we can identify subgroups of the population with certain covariates. Importantly, the SCE enables us to approximate treatment effects for patients with systematically missing data at test time by assigning them to similar subgroups of the population on the basis of their covariates.

### 3.2. Problem Formulation

**Figure 2:**
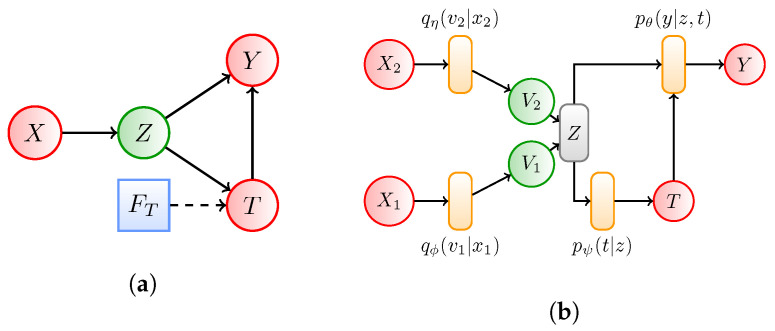
(**a**) Influence diagram of the Cause-Effect Information Bottleneck (CEIB) . Red and green circles correspond to observed and latent random variables respectively, while blue rectangles represent interventions. We identify a low-dimensional representation *Z* of covariates *X* to estimate the effects of an intervention on outcome *Y* where data are systematically missing at test time. (**b**) Graphical illustration of the CEIB. Orange rectangles represent deep networks parameterising the random variables. Our encoder networks *q_ϕ_*(*v*_1_|*x*) and *q_η_*(*v*_2_|*x*) try to minimise the first two terms in Equation (10), while the decoder *p_θ_(y|t,z)* tries to minimise the last term in Equation (10).

Let $X=(X_{1},X_{2})$ denote the set of patient covariates (confounders) based on which we would like to estimate treatment effects. During training we assume that all covariates $X\in R^{d}$ can be observed. These correspond to, for instance, the measurements of a set of patients participating in a medical study, where dimension *d* is large. Outside the study at test time however, we assume covariates $X_{1}$ are not observable, e.g., due to the expensive data acquisition process. This corresponds to having a fixed set of features that are systematically missing for a subset of patients during testing. Let Y∈R denote the outcomes following treatments *T*. For simplicity and ease of comparison with prior methods on existing benchmarks, we consider treatments *T* that are binary, but our method is applicable for any general *T*. We assume that all confounders are measurable (also known as strong ignorability) during training and testing. That is, the set of confounders is measurable across all patients and do not comprise the systematically missing covariates during testing. The causal graph corresponding to our model is shown in Figure 2a. Finally, we assume that low-dimensional *Z* does not capture any post-treatment variables, that may otherwise bias predictions. Under these assumptions, estimating the ACE requires computing a distribution Y|Z,T, provided *Z* is a sufficient covariate. In what follows, we use the IB to learn such a sufficient covariate that allows us to approximate the distribution Y∣Z,T in Figure 2a.

### 3.3. IB Method for Performing a Sufficient Reduction of the Covariate

We now develop an extended formulation of the Information Bottleneck namely CEIB, for estimating the effects of an intervention. Specifically, the new IB formulation enables us to learn a low-dimensional, interpretable compression of the relevant information during training such that we obtain a sufficiently reduced covariate *Z*. Based on this information, we can subsequently infer treatment effects where covariates may be systematically missing at test time. To do so, we consider the following adapted parametric form of the IB,

(10) $$\begin{array}{c} \max_{\phi ,\theta ,\psi ,\eta }-I_{\phi }\left(V_{1};X_{1}\right)-I_{\eta }\left(V_{2};X_{2}\right)+\lambda I_{\phi ,\theta ,\psi ,\eta }\left(Z;\left(Y,T\right)\right), \end{array}$$

where $V_{1}$ and $V_{2}$ are compressed discrete representations of the covariates, $Z=(V_{1},V_{2})$ is a concatenation of $V_{1}$ and $V_{2}$ and *I* represents the mutual information parameterised by ϕ, ψ, θ, and η respectively. We assume a parametric form of the conditionals $q_{\phi }\left(v_{1}|x\right)$, $q_{\eta }\left(v_{2}|x\right)$, $p_{\theta }\left(y|t,z\right)$, $p_{\psi }\left(t|z\right)$. The modified IB criterion in Equation (10) reflects that we would like to obtain an optimal compression *Z* of *X* while simultaneously retaining information about *Y* (given *T*). The first two terms of our new IB formulation are given by:

(11) $$\begin{array}{cc} I_{\phi }\left(V_{1};X_{1}\right) & =D_{KL}(q_{\phi }\left(v_{1}|x_{1}\right)p\left(x_{1}\right)||p\left(v_{1}\right)p\left(x_{1}\right))=E_{p(x_{1})}D_{KL}(q_{\phi }\left(v_{1}|x_{1}\right)||p\left(v_{1}\right)) \end{array}$$

(12) $$\begin{array}{cc} I_{\eta }\left(V_{2};X_{2}\right) & =D_{KL}(q_{\eta }\left(v_{2}|x_{2}\right)p\left(x_{2}\right)||p\left(v_{2}\right)p\left(x_{2}\right))=E_{p(x_{2})}D_{KL}(q_{\eta }\left(v_{2}|x_{2}\right)||p\left(v_{2}\right)), \end{array}$$

while the last term has the form:

(13) $$\begin{array}{cc} I_{\phi ,\theta ,\psi ,\eta }\left(Z;\left(Y,T\right)\right) & \geq E_{p(x,y,t)}E_{p_{\phi ,\eta }\left(z|x\right)}\left[\log p_{\theta }\left(y|t,z\right)+\log p_{\psi }\left(t|z\right)\right]+H{\left(y,t\right)}^{1}, \end{array}$$

where $H\left(y,t\right)=-E_{p(y,t)}\log p\left(y,t\right)$ is the entropy of (y,t). The lower bound follows from the fact that the mutual information between *Z* and Y,T can be expressed as a sum of the expected value of $\log p_{\theta }\left(y|t,z\right)+\log p_{\psi }\left(t|z\right)$, entropy H(y,t) and two KL-divergences, which are by definition non-negative [36]. Specifically, in Equation (13) we bound I(Z;Y) using the result from [36] such that we no longer require the original IB conditional independence assumption described in Section 2.4. Importantly, optimising the criterion in Equation (10) enables us to learn a sufficiently reduced covariate *Z* which can be used to accurately estimate the ACE using Equation (8). Unlike other approaches for inferring treatment effects, the Lagrange parameter λ in the IB formulation in Equation (10) allows us to adjust the degree of compression, which, in this context, enables us to learn a sufficient statistic *Z*. In particular, adjusting λ enables us to explore a range of such representations from having a completely insufficient covariate where $Y⊥\;⊥F_{T}|\left(Z,T\right)$, to a completely sufficient compression of confounding where $Y⊥\;⊥F_{T}|\left(Z,T\right)$ — a property crucial for auditing and interpreting the resulting predictions. Note that in general, we are free to choose a suitable parametric form in order to optimise Equation (10). In what follows, we describe how to do so with neural networks.

### 3.4. Implementation of the IB Method

We now describe how to implement and optimise the criterion in Equation (10). The proposed architecture of our model is illustrated in Figure 2b. I(X;V1) and I(X;V2) are parameterised by two neural networks (also known as encoder networks). The encoder networks try to minimise the first two terms in Equation (10). I(Z;(Y,T)) is parameterised by another neural network (known as the decoder network). The decoder network tries to minimise the last term of the loss in Equation (10).

For our encoder architectures, we specifically compress *X* to obtain discrete latent representations $V_{1}$ and $V_{2}$ of the covariate information. To do so, we make use of the Gumbel softmax reparameterisation trick [41] to draw samples *Z* from a categorical distribution with probabilities π. Here,

(14) $$\begin{array}{c} z=\mathrm{one}\_\mathrm{hot}(\arg\max_{i}\left[g_{i}+\log\pi_{i}\right]), \end{array}$$

where one_hot refers to a one-hot feature encoding and $g_{1},g_{2},\ldots ,g_{k}$ are samples drawn from Gumbel(0,1). The softmax function is used to approximate the argmax in Equation (14), and generate *k*-dimensional sample vectors $w\in \Delta^{k-1}$, where

(15) $$\begin{array}{c} w_{i}=\frac{\exp(\left(\log\left(\pi_{i}\right)+g_{i}\right)/\tau )}{\sum_{j=1}^{k}\exp\left(\left(\log\left(\pi_{j}\right)+g_{j}\right)/\tau \right)},i=1,\ldots ,k. \end{array}$$

and τ is the softmax temperature parameter.

For our decoder network, we use an architecture similar to the TARnet [6], where we replace conditioning on high-dimensional covariates *X* with conditioning on reduced covariate *Z*. We can thus formulate the conditionals as,

(16) $$\begin{array}{c} p_{\psi }\left(t|z\right)=\mathrm{Bern}\left(\sigma \left(f_{1}\left(z\right)\right)\right)\;\\ p_{\theta }\left(y|t,z\right)=N\left(\mu =\hat{\mu },\varsigma^{2}=\hat{s}\right), \end{array}$$

with logistic function σ(·), and outcome *Y* given by a Gaussian distribution parameterised with a TARnet with $\hat{\mu }=tf_{2}\left(z\right)+\left(1-t\right)f_{3}\left(z\right)$. Note that the terms $f_{k}$ correspond to neural networks. While distribution p(t|z) is included to ensure the joint distribution over treatments, outcomes and covariates is identifiable, in practice, our goal is to approximate the effects of a given *T* on *Y*. Hence, we train our model in a teacher forcing fashion by using the true treatment assignments *T* from the data and fixing the *T*s at test time.

Overall, by using the Gumbel softmax reparameterisation trick to obtain a discrete representation of relevant information, we can learn equivalence classes among patients based on which we can compute the SCE for each group using sufficient covariate *Z* via Equation (9). This has an important implication. Specifically, it means that during training our method reduces covariates *X* to a sufficiently compressed covariate *Z*. If a subset $X_{1}$ of *X* is unavailable after training, we can still use the learnt *Z* to compute the ACE during testing, since the *m*-graphs associated with Figure 2a no longer contain only random variables and hence become degenerate. Thus at test time, we can subsequently assign an example with missing covariates to its relevant equivalence class. Here, computing the SCE allows us potentially to tailor treatments to specific groups based on *Z* rather than an entire population– an important aspect in healthcare where patients are typically heterogeneous. Based on the SCE, we can also compute the population-level effects of an intervention via the ACE from Equation (8). In the absence of the latent compression via CEIB and the discrete representation of relevant information, it would not be possible to transfer knowledge from examples with complete information to cases with systematically missing information, since estimating treatment effects would require integrating over all the covariates—an infeasible task in high dimensions.

## 4. Experiments

The lack of ground truth in real world data makes evaluating causal inference methods a difficult problem. To overcome this issue, existing approaches typically consider using semi-synthetic data sets where outcomes and treatment assignments are fully known, or randomised control trial experiments. Our goal is to demonstrate the ability of CEIB to accurately infer treatment effects, while simultaneously learning a low-dimensional, interpretable representation of confounding in cases where covariate information is systematically missing at test time. For this purpose, we consider a task-based on a randomised control experiment and a second semi-synthetic task for treating low birth-weight twins. Additionally, we demonstrate the performance of our approach on a real high-dimensional task for managing and treating sepsis. In our experiments, we report both the SCE and ACE values for this purpose.

### 4.1. Infant Health and Development Program

The Infant Health and Development Program (IHDP) [45,46] is a randomised control experiment assessing the impact of educational intervention on the outcomes of pre-mature low birth-weight infants born between 1984 and 1985. Measurements from children and their mothers were collected for studying the effects of childcare and home visits from a trained specialist on test scores. The study contains information about the children and their mothers/caregivers. Data on children includes sex, birth weight, head circumference and other health indices. Information about mothers includes maternal age and race, as well as educational achievement. Together, these data comprise our data set, denoted as *X*. Treatments or interventions *T* are binary indicators corresponding to participation in the IHDP child development centres, while outcomes *Y* correspond to the IQ-test score measured following intervention.

Like Hill [46], features and treatment assignments are extracted from the real world clinical trial, and selection bias is introduced in the data by artificially removing a non-random portion of the treatment group, in particular children with non-white mothers. In total, the resulting data set then consists of 747 subjects (139 treated, 608 control), each represented by 26 covariates measuring the properties of the child and their mother. We subsequently divide this data set into 60/10/30% training/validation/test sets. For our setup, we use encoder and decoder architectures with 3 hidden layers and train the model using Adam as an optimiser with a learning rate of 0.001. We train our model with four 3-dimensional Gaussian mixture components, although our method can be applied, without loss of generality, to any number of dimensions. We compare the performance of CEIB for predicting the ACE against several existing baselines, first for the case where no covariates are systematically missing at test time, and subsequently by considering the best performing baselines for the case where covariates are systematically missing during testing. Specifically, we consider the following baselines: OLS-1 is a least squares regression; OLS-2 uses two separate least squares regressions to fit the treatment and control groups respectively; TARnet is a feedforward neural network from Shalit et al. [47]; KNN is a *k*-nearest neighbours regression; RF is a random forest; BNN is a balancing neural network [6]; BLR is a balancing linear regression [6]; BART is a Bayesian Additive Regression Tree model [48,49]; CFRW is a counterfactual regression that uses the Wasserstein distance [47].

**Experiment 1:** In the first experiment, we compared the performance of CEIB for estimating the ACE against the baselines when using the complete set of measurements at test time. In Table 1a we report the difference between the true ACE and our CEIB estimate since we have de-randomised the data. We repeat this on multiple data splits. Evidently, CEIB outperforms existing approaches. To demonstrate that we can transfer the relevant information to cases where covariates are systematically missing at test time, we artificially excluded n=3 covariates that have a moderate correlation with ethnicity at test time. We compute the ACE and compare this to the performance of the three best performing baselines namely, TARnet, CFRW and BART applied to the reduced set of covariates (Table 1b). If we extend this to the extreme case of removing 8 covariates at test time, the out-of-sample error in predicting the ACE increases to 0.29 +/− 0.02. Thus CEIB achieves the highest predictive performance for both in-sample and out-of-sample predictions, even with systematically missing covariates. Overall, these results largely match our expectations since prediction accuracy for all the approaches decreases when covariates are systematically missing at test time. We attribute the difference in performance to the way in which CEIB uses covariate information when making predictions. Unlike any of the other methods, CEIB specifically extracts only the information that is relevant for making predictions, and uses this to learn a suitable representation of confounding to infer treatment effects.

**Figure 3:**
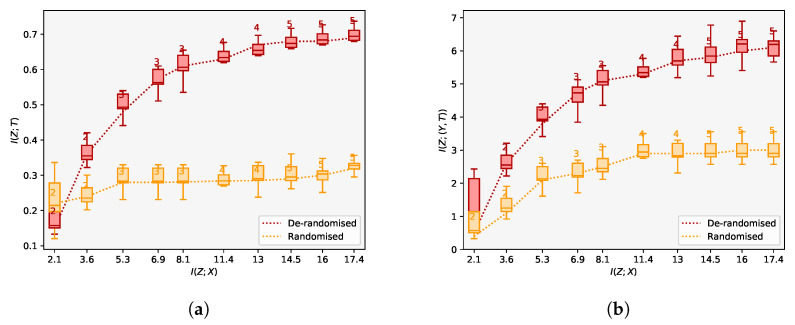
(**a**) Information curves for *I(Z; T)* and (**b**) *I(Z; (Y, T))* with de-randomised and randomised data respectively. When the data are randomised, the value of *I(Z; T)* is close to zero. The differences between the curves illustrates confounding. When data are de-randomised, we are able to estimate treatment effects more accurately by accounting for this confounding.

**Experiment 2:** Building on Experiment 1, we perform an analysis of the latent space of our model to assess whether we learn a sufficiently reduced covariate. We use the IHDP data set as before, but this time consider both the data before introducing selection bias (analogous to a randomised study), as well as after introducing selection bias by removing a non-random proportion of the treatment group as before (akin to a de-randomised study). We plot the information curves illustrating the number of latent dimensions required to reconstruct the output for the terms I(Z;(Y,T)) and I(Z,T) respectively for varying values of λ. These results are shown in Figure 3a,b. Theoretically, we should be able to examine the shape of the curves to identify whether a sufficiently reduced covariate has been obtained. In particular, when a study is randomised, the sufficient covariate *Z* should have no impact on the treatment *T*. In this case, the mutual information I(Z,T) should be approximately zero and the curve should remain flat for varying values of I(Z,X). This result is confirmed in Figure 3a. The information curves in Figure 3b additionally demonstrate our model’s ability to account for confounding when predicting the overall outcomes: when data are de-randomised, we are able to reconstruct treatment outcomes more accurately. Specifically, the point at which each of the information curves saturates is the point at which we have learnt a sufficiently reduced covariate based on which we can infer treatment effects. Overall, the results from Figure 3a,b highlight another benefit of using CEIB for estimating treatment outcomes: in particular, by adjusting the Lagrange parameter λ, CEIB allows for a task-dependent adjustment of the latent space. This adjustment allows one to explore a full range of solutions across the information curve, from having a completely insufficient covariate to a completely sufficient compression of the covariates where the information curve saturates. In the absence of the IB objective, this is not possible. Overall, we are able to learn a low-dimensional representation that is consistent with the ethnicity confounder. By conditioning on this representation, we can thus account for its effects when predicting treatment outcomes.

**Figure 4:**
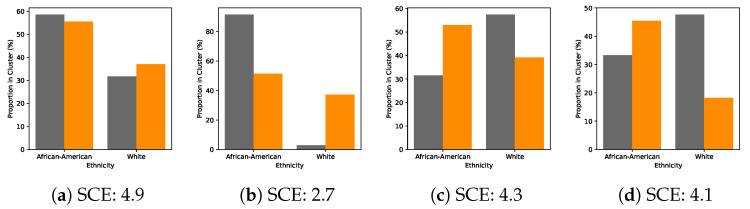
Illustration of the proportion of major ethnic groups within the four clusters. Grey and orange indicate de-randomised and randomised data respectively. For better visualisation, we only report the two main clusters which include the majority of all patients. The first cluster in (**a**) is a neutral cluster. The second cluster in (**b**) shows an enrichment of information in the African-American group. Clusters 3 and 4 in (**c**,**d**) respectively, show an enrichment of information in the White group. Overall, the clusters exhibit a distinct structure with respect to the known ethnicity confounder. Moreover, each of the clusters is associated with different Specific Causal Effect (SCE) values. In particular, the second cluster has a lower SCE which suggests that educational intervention for these members has less of an impact on outcomes—a result consistent with our de-randomisation strategy.

**Figure 5:**
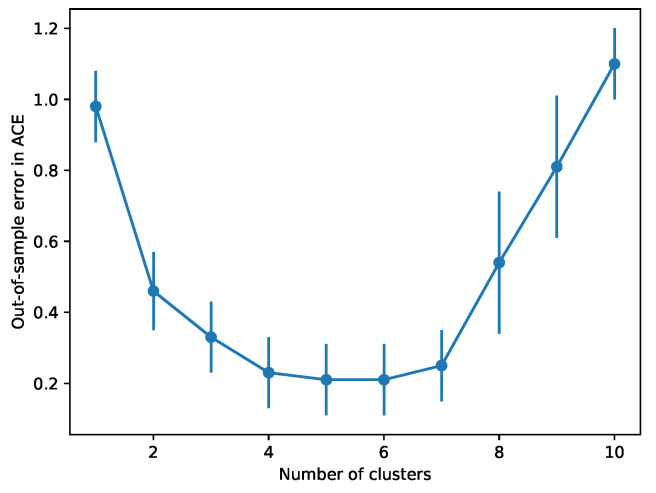
Out-of-sample error in ACE with a varying number of clusters.

We also analysed the discretised latent space by comparing the proportions of ethnic groups of test subjects in each cluster in the de-randomised setting. These results are shown in Figure 4 where we plot a hard assignment of test subjects to clusters on the basis of their ethnicity. Evidently, the clusters exhibit a clear structure with respect to ethnicity. In particular, Cluster 2 in Figure 4b has a significantly higher proportion of non-white members in the de-randomised setting. The discretisation also allows us to calculate the SCE for each cluster. In general, Cluster 2 tends to have a lower SCE than the other groups. This is consistent with how the data were de-randomised, since we removed a proportion of the treated instances with non-white mothers. Conditioning on this kind of information is thus crucial to be able to accurately assess the impact of educational intervention on test scores. Finally, we assess the error in estimating the ACE when varying the number of mixture components in Figure 5. When the number of clusters is larger, the clusters get smaller and it becomes more difficult to reliably estimate the ACE since we average over the cluster members to account for partial covariate information at test time. Here, model selection is made by observing where the error in estimating the ACE stabilises (anywhere between 4 and 7 mixture components).

### 4.2. Binary Treatment Outcome on Twins

As a second application, we make use of data about twin births in the USA between 1989 and 1991 [50]. Here, treatment T=1 is a binary indicator of being the heavier twin at birth, while outcome *Y* corresponds to the mortality within a year after birth. Since mortality is rare, we consider only same sex twins with weights less than 2 kg, which results in 11,984 pairs of twins. Each twin has a set of 46 covariates comprising our dataset *X*. These covariates include information about parents of the twins such as their level of education, race, incidence of renal disease, diabetes, smoking etc., as well as whether the birth took place in hospital or at home and the number of gestation weeks prior to birth.

**Figure 6:**
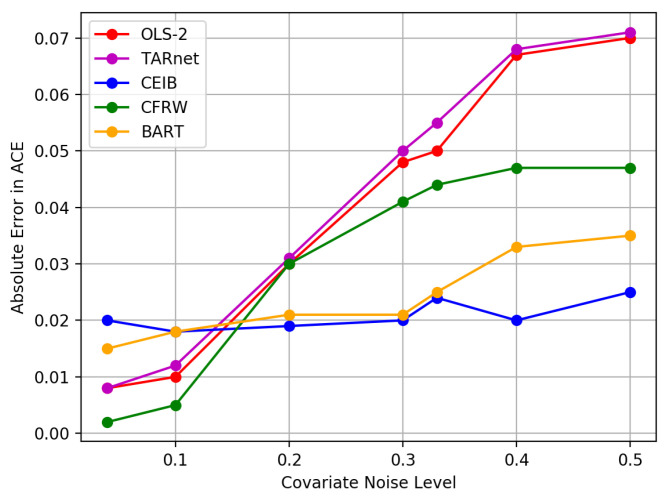
Absolute error in ACE estimation for Twins task. CEIB outperforms baselines over varying levels of covariate noise.

To simulate an observational study, we selectively hide one of the twins. Treatments *T* are allocated based on a single variable which is highly correlated with the outcome: GESTAT10, the number of gestation weeks prior to birth. This has values from 0 to 9 that correspond to the weeks of gestation before birth, i.e., birth before 20 weeks gestation, 20-27 weeks of gestation, etc. Analogous to [12], we set treatment to $t|x,z∼\mathrm{Bern}(\sigma \left(w_{o}^{⊤}x+w_{h}\left(z/10-0.1\right)\right))$ for $w_{o}∼N\left(0,0.1I\right),w_{h}∼N\left(5,0.1\right)$, where *z* is GESTAT10 and *x* are the 45 remaining covariates. We compared the performance of CEIB to TARnet, OLS 2, BART, CFRW in terms of the errors in estimating the ACE. These results are summarised in Figure 6.

Overall, CEIB outperforms each of the baselines under varying levels of covariate noise. We attribute this to the fact that CEIB extracts only the information that is relevant for making predictions (via the IB criterion) in order to draw inferences about the effects of interventions.

### 4.3. Sepsis Management

We also illustrate the performance CEIB on the real-world task of managing and treating sepsis. Sepsis is one of the leading causes of mortality within hospitals and treating septic patients is highly challenging, since outcomes vary with interventions and there are no universal treatment guidelines. For this experiment, we make use of data from the Multiparameter Intelligent Monitoring in Intensive Care (MIMIC-III) database [51]. We focus specifically on patients satisfying Sepsis-3 criteria (16 804 patients in total). For each patient, we have a 48-dimensional set of physiological parameters including demographics, lab values, vital signs and input/output events, where certain covariates are systematically missing. We denote this set as *X*. Our outcomes *Y* correspond to the odds of mortality, while we binarise medical interventions *T* according to whether or not a vasopressor is administered. The data set is divided into 60%/20%/20% into training/validation/testing sets. We train our model with six 4-dimensional Gaussian mixture components and analysed the information curves and cluster compositions respectively.

**Figure 7:**
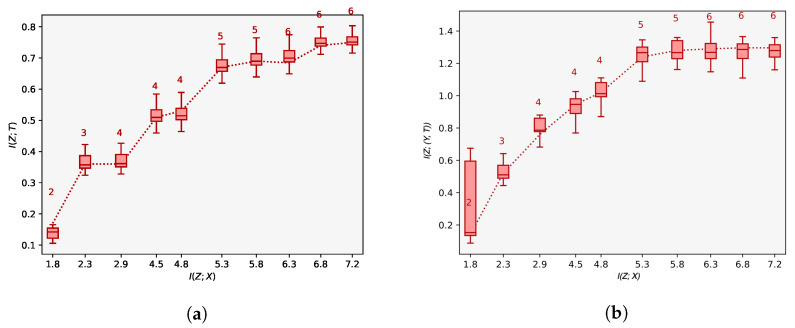
Subfigures (**a**,**b**) illustrate the information curve *I(Z; T)* and *I(Z; (Y, T))* for the task of managing sepsis. We perform a sufficient reduction of the covariates to 6-dimensions and are able to approximate the ACE on the basis of this.

**Figure 8:**
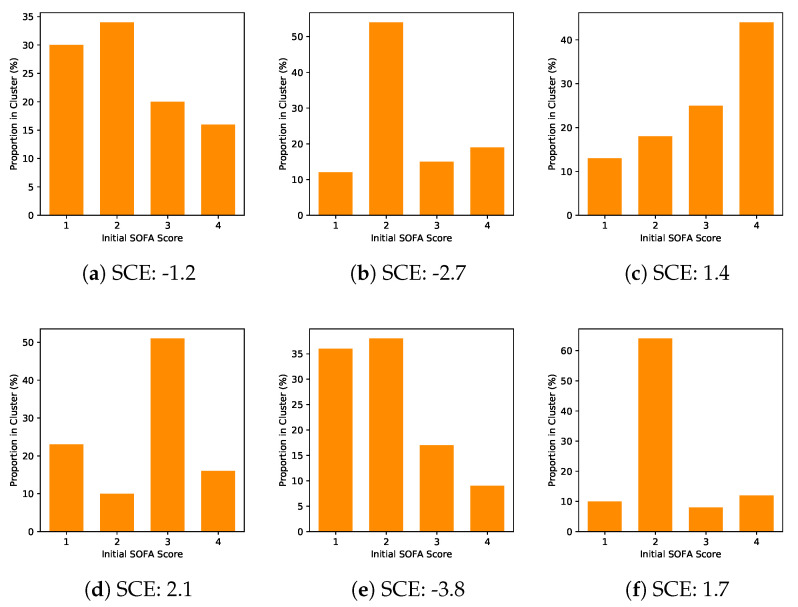
Proportion of initial Sequential Organ Failure Assessment (SOFA) scores in each cluster. The variation in initial SOFA scores across clusters suggests that it is a potential confounder of odds of mortality when managing and treating sepsis.

The information curves for I(Z;T) and I(Z;(Y,T)) are shown in Figure 7a,b respectively. We observe that we can perform a sufficient reduction of the high-dimensional covariate information to between four and six dimensions while achieving high predictive accuracy of outcomes *Y*. Since there is no ground truth available for the sepsis task, we do not have access to the true confounding variables. However, we can perform an analysis on the basis of the clusters obtained over the latent space. Here, we see that we can characterise the patients in each cluster according to their initial SOFA (Sequential Organ Failure Assessment) scores. SOFA scores range between 1 and 4 and are used to track a patient’s stay in hospital. In Figure 8, we observe clear differences in cluster composition relative to the SOFA scores. Clusters 2, 5 and 6 tend to have higher proportions of patients with lower SOFA scores, while Clusters 3 and 4 have larger proportions of patients with higher SOFA scores. This result suggests that a patient’s initial SOFA score is potentially a confounder when determining how to administer subsequent treatments and predicting their odds of in-hospital mortality. This is consistent with medical studies such as Medam et al. [52], Studnek et al. [53] where authors indicate that high initial SOFA scores were likely to impact on their overall chances of survival and treatments administered in hospital.

While we cannot quantify an error in estimating the ACE since we do not have access to the counterfactual outcomes, we can still compute the ACE for the sepsis management task. Here, we specifically observe a negative ACE value. This means that in general, treating patients with vasopressors reduces the chances of mortality in comparison to not treating patients with vasopressors. Overall, performing such analyses for tasks like sepsis may shed light on what information is relevant for making predictions and reasoning about the effects of medical intervention. In turn, this may assist in establishing potential therapy guidelines for better decision-making.

## 5. Discussion

### 5.1. Low-Dimensional, Interpretable Representations of Confounding

Because CEIB is explicitly constrained to extract only the information that is relevant for making predictions about outcomes, it is capable of learning a low-dimensional representation of confounding, using which we can base our predictions. Specifically, by introducing a discrete clustering structure in the latent space of our model, we can easily inspect and interpret the confounding effects. For the IHDP experiment, this means we can learn a low-dimensional representation of the known ethnicity confounder and account for its effects when predicting treatment outcomes. Earlier methods such as Louizos et al. [12] use higher dimensional representations (in the order of 20 dimensions) to account for these effects yet make less accurate predictions. A possible explanation for this is that the true confounding effect is misrepresented. Consequently, modelling the task using the IB model alleviates this issue. Similarly for the task of treating septic patients in ICU, we identified a low-dimensional latent space of 6 dimensions for predicting the odds of mortality, where clusters exhibited a distinct structure with respect to a patient’s initial SOFA score. Importantly, across all tasks, the low-dimensional representation allows us to accurately identify confounders whilst retaining model interpretability.

### 5.2. Accurate Estimation of the Causal Effect with Systematically Missing Covariates during Testing

Unlike earlier approaches, CEIB can deal with systematically missing data during test time through the introduction of a discretised latent space via the Gumbel softmax reparameterisation trick. Based on this representation, we can learn equivalence classes among patients such that the approximate the effects of treatments can be computed.

### 5.3. State-of-the-Art ACE Predictions That Are Robust against Confounding

Across the IHDP dataset, we see that predictions of the ACE are considerably more accurate than existing approaches. In the IHDP case, we see reductions in the error in estimating the ACE up to 0.58 for in-sample predictions. This performance is sustained when making out-of-sample predictions we see error reductions of between 0.04 and 0.73 in comparison with existing methods. Overall, we attribute this increase in performance directly to the fact that CEIB extracts only the information that is relevant for making predictions. Proxy-based approaches such as Louizos et al. [12] do not explicitly trade off learning meaningful representations of confounders and achieving accurate predictions. In contrast, we can explicitly inspect the information curves in Figure 3b and adjust compression parameter λ to expose a relevant representation of confounding. If we set λ=13 in accordance to Figure 3b, we require only a 4-dimensional representation to adequately account for and uncover the true confounding effect *Z* (as shown in Figure 4b). This produces more accurate predictions as a result.

## 6. Conclusions

We have presented a novel approach to estimate causal relationships with systematically missing covariates at test time. This is an important problem particularly in healthcare, since doctors frequently have access to certain routine measurements, but may have difficulty acquiring others such as genotype information. For this purpose, we analysed the role of a sufficient covariate in the context of the IB framework. This included introducing a discrete latent space to facilitate transferring knowledge to cases where information was systematically missing—a task that is otherwise infeasible in high dimensions. In doing so, we could estimate the causal effect if parts of the covariates are missing during test time, while accounting for confounding. In contrast to previous methods, the compression parameter λ in the IB framework allows for a task-dependent adjustment of the latent dimensionality. Our extensive experiments showed that our method outperforms state-of-the-art approaches on multiple synthetic and real world datasets. More broadly, since handling systematic missingness is a highly relevant problem in healthcare, we view this as step towards improving these systems on a larger scale. Directions for future work include adapting our approach to infer other causal concepts such as the Effect of Treatment on Treated (ETT) for instance, through the use of instrumental variables; as well as relating the model to existing double robustness techniques and propensity scoring methods.

## Figures and Tables

**Table:** (**a**)

Method	$\epsilon_{\mathrm{ACE}}^{\mathrm{within}-s}$	$\epsilon_{\mathrm{ACE}}^{\mathrm{out}-\mathrm{of}-s}$
OLS-1	0.73±0.04	0.94±0.06
OLS-2	0.14±0.01	0.34±0.02
KNN	0.14±0.01	0.79±0.05
BLR	0.72±0.04	0.93±0.05
TARnet	0.26±0.01	0.28±0.01
BNN	0.37±0.03	0.42±0.03
RF	0.73±0.05	0.96±0.06
BART	0.23±0.01	0.33±0.02
CEVAE	0.34±0.01	0.46±0.02
CFRW	0.25±0.01	0.27±0.01
**CEIB**	0.11±0.01	0.21±0.01

**Table:** (**b**)

Method	$\epsilon_{\mathrm{ACE}}^{\mathrm{out}-\mathrm{of}-s}$
TARnet	0.34±0.01
CFRW	0.49±0.02
BART	0.39±0.02
**CEIB**	0.23±0.01
